# Exploiting the miniature inverted-repeat transposable elements insertion polymorphisms as an efficient DNA marker system for genome analysis and evolutionary studies in wheat and related species

**DOI:** 10.3389/fpls.2022.995586

**Published:** 2022-09-02

**Authors:** Benjamin Ewa Ubi, Yasir Serag Alnor Gorafi, Beery Yaakov, Yuki Monden, Khalil Kashkush, Hisashi Tsujimoto

**Affiliations:** ^1^Molecular Breeding Laboratory, Arid Land Research Center, Tottori University, Tottori, Japan; ^2^Department of Biotechnology, Ebonyi State University, Abakaliki, Abakaliki, Ebonyi, Nigeria; ^3^International Platform for Dryland Research and Education, Tottori University, Tottori, Japan; ^4^Agricultural Research Corporation, Wad Medani, Sudan; ^5^French Associates Institute for Agriculture and Biotechnology of Drylands, Jacob Blaustein Institutes for Desert Research, Ben-Gurion University of the Negev, Beer-Sheva, Israel; ^6^Graduate School of Environmental and Life Science, Okayama University, Okayama, Japan; ^7^Department of Life Sciences, Ben-Gurion University, Beer-Sheva, Israel

**Keywords:** transposable elements, mite, DNA markers, Dryland, wheat, genome analysis

## Abstract

Transposable elements (TEs) constitute ~80% of the complex bread wheat genome and contribute significantly to wheat evolution and environmental adaptation. We studied 52 TE insertion polymorphism markers to ascertain their efficiency as a robust DNA marker system for genetic studies in wheat and related species. Significant variation was found in miniature inverted-repeat transposable element (MITE) insertions in relation to ploidy with the highest number of “full site” insertions occurring in the hexaploids (32.6 ± 3.8), while the tetraploid and diploid progenitors had 22.3 ± 0.6 and 15.0 ± 3.5 “full sites,” respectively, which suggested a recent rapid activation of these transposons after the formation of wheat. Constructed phylogenetic trees were consistent with the evolutionary history of these species which clustered mainly according to ploidy and genome types (SS, AA, DD, AABB, and AABBDD). The synthetic hexaploids sub-clustered near the tetraploid species from which they were re-synthesized. Preliminary genotyping in 104 recombinant inbred lines (RILs) showed predominantly 1:1 segregation for simplex markers, with four of these markers already integrated into our current DArT-and SNP-based linkage map. The MITE insertions also showed stability with no single excision observed. The MITE insertion site polymorphisms uncovered in this study are very promising as high-potential evolutionary markers for genomic studies in wheat.

## Introduction

Wheat (*Triticum* spp.) is a cereal crop of major importance globally which provides about 20% of the calories consumed by man (FAO, 2015); and is a foremost source of vegetable protein in the human diet relative to other major cereal crops such as maize or rice (Wheat-Wikipedia, n.d.). The increased production of cereals such as wheat is urgently needed to meet the demand gap for global food supply by the year 2050 (Tester and Langridge, 2010) when 60%–70% increase in the food production is required to feed the projected rapid increase in population (Silva, 2018). Allohexaploid bread wheat (*Triticum aestivum*, 2*n* = 6x = 42, AABBDD) is of relatively recent origin, having evolved ~8,500 years ago following two separate interspecific hybridization events involving three diploid donor species (*n* = 7; Leach et al., 2014). First, the tetraploid (pasta) wheat (*T. turgidum* L. ssp. *durum*; 2*n* = 4x = 28, AABB) arose from a hybridization event between *T. urartu* (2*n* =2x = 14, AA) and another yet unknown wild diploid relative of *Aegilops speltoides* (2*n* = 2x = 14, BB) about 0.5 million years ago (Dvorak and Akhunov, 2005; Yaakov et al., 2012; Ogbonnaya et al., 2013). The cultivated *T. turgidum* then crossed with *Aegilops tauschii*, a wild diploid relative (2*n* = 2x = 14, DD) resulting in the modern day allohexaploid bread wheat with its 42 chromosomes distributed in the A, B and D homoeologous sets contributed by the three diploid progenitors.

Several phylogenetic studies have been undertaken over the years to characterize the taxonomic relationships between members of the *Triticum* (wheat)**—***Aegilops* complex. Polymorphic transposable element (TE) insertion sites have recently been shown to be a promising tool for the analysis of the phylogenetic relationships in wheat (Konovalov et al., 2010; Yaakov et al., 2012, 2013). TE-based DNA marker systems showed relative superiority over other marker systems in resolving phylogenetic relationships due largely to their high variability and informativeness (Konovalov et al., 2010), but such relative superiority might be dependent on the TE activity level during the course of evolution and their ability to generate insertion site polymorphisms.

In plant species, DNA-marker systems based on different TEs [e.g., miniature inverted-repeat transposable element (MITEs), long terminal repeat (LTR) elements, CACTA transposons, etc.] have been exploited for various genetic studies (Hirsch and Springer, 2017; Morata et al., 2018; Quesneville, 2020). MITEs are thought to be a peculiar type of non-autonomous Class II TE, activated by transposases encoded by their related autonomous elements, which might have enhanced their rapid amplification to potentially generate high copy numbers, though the mechanism of their amplification is yet unknown (Casacuberta and Santiago, 2003; Fattash et al., 2013; Chen et al., 2014). However, recent efforts by Castanera et al. (2021) suggested a replicative mechanism underlying the amplification dynamics of MITEs. MITEs typically have relatively short sequences (generally < 600 bp), large copy numbers, an AT-rich sequence, contain terminal inverted repeats (TIRs) and flanked by two short direct repeats referred to as target site duplications (TSD; Casacuberta and Santiago, 2003; Yaakov et al., 2012, 2013; Fattash et al., 2013; Chen et al., 2014; Li et al., 2014). Plant MITEs are categorized into two main families, *Habinger*/*Tourist*-like and *Mariner*/*Stowaway*-like, besides several other minor families. Recent genome-wide analysis of MITEs based on genome drafts of four wheat and related species [polyploids: *T. aestivum* and *T. turgidum* ssp. *dicoccoides*, and diploids: *Ae*. *tauschii* and *T*. *urartu*] involving 239,126 retrieved MITE insertions showed the *Stowaway-like* superfamily as the most abundant (83.4%) in the wheat genome, followed by *Tourist-like* superfamily (4.9%), *Mutator* (2.7%), with 8.9% being unknown; and novel wheat-unique family named “Inbar” belonging to the Stowaway-like superfamily, was also identified in this large-scale study (Keidar-Friedman et al., 2018). The relative on the most abundant MITEs in the wheat genome was found to be the *Stowaway*-like family (62.6%), followed by the *Tourist*-like family (12.1%), while all other families were not found. The relatively small size and high copy numbers of MITEs facilitate their invasiveness and frequent insertion into genomic regions such as promoters, untranslated regions, introns or coding sequences of genes; though they are thought to predominate in the non-coding regions of eukaryotic genes (Han and Wessler, 2010; Li et al., 2014). MITEs are often inserted within gene-rich euchromatic regions and frequently found associated with genes (Wessler et al., 1995; Yasuda et al., 2013; Chen et al., 2014).

Insertion site polymorphisms generated by MITEs can be a valuable molecular marker system for exploitation in various genetic and breeding studies (Monden et al., 2009; Shirasawa et al., 2012; Yaakov et al., 2012; Yaakov and Kashkush, 2012; Mondal et al., 2014; Wang et al., 2020). The simple inheritance of MITE insertion site polymorphisms, their low cost of detection, their dominant and/or co-dominant nature, and their ability to generate polymorphisms even between closely related genomes makes them suitable DNA markers for studying genetic diversity, association mapping and trait mapping.

In this study, 52 TE insertion polymorphism markers selected from 13 *Stowaway*-like MITE families (Yaakov et al., 2012) were investigated to ascertain their efficiency for exploitation as a robust DNA marker system for genetic diversity, linkage analysis and evolutionary studies in wheat. Furthermore, our investigation of the excision frequency of 16 polymorphic MITE markers in one of the parents of our mapping population (*T. aestivum* cv. Chinese Spring) revealed a putative stable inheritance, which is promising for genetic linkage analysis, evolutionary studies and as a useful tool for wheat molecular breeding.

## Materials and methods

### Plant materials and DNA extraction

In this study, we used 17 *Triticum* and *Aegilops* accessions (Table 1). In the initial amplification and isolation of MITE fragments, we used five accessions that are indicated by bold interface in Table 1. After the initial screening and isolation, we used an additional 12 accessions to study the variation in MITE insertions and phylogenetic analysis. *T. carthlicum* and *Ae. tauschii* are the parents of SHW ABD4 (Table 1). Syn.72 is an amphidiploid between Langdon and *Ae. tauschii* acc. PI508262. MSD-original #1, MSD-2 waxless and MSD-5 heat tolerant are multiple synthetic derivative lines selected from the original multiple synthetic derivatives population, the waxless and heat-tolerant subpopulations, respectively (Tsujimoto et al., 2015; Elbashir et al., 2017). The genomic DNA of all plants was isolated from fresh young leaf tissue (~0.5 g) using a modified CTAB-based miniprep extraction method. Briefly, 0.5 g of freshly harvested young wheat leaf samples were collected into a 2-ml eppendorf tube (frozen in liquid nitrogen) and kept at −80°C until ground into a fine powder (under liquid nitrogen) using the MicroMixer. A 1-ml pre-heated (65°C) 3% CTAB extraction buffer [containing 3% (w/v) CTAB, 1.4 M NaCl, 0.1 M Tris–HCl (pH 8.0), 0.02 M EDTA (pH 8.0), and 1% (v/v) *β*-Mercaptoethanol] was added to the ground frozen tissue and mixed briefly by tube inversions; and then tissue homogenization was carried out in a water bath set at 55°C for 30 min. Following tissue homogenization, 800 μl of Chloroform: Isoamyl alcohol (CI, 24:1) was added and gently but thoroughly mixed for 5 min, before centrifugation at 5,000 rpm for 5 min at room temperature. The supernatant was transferred to a new tube; and DNA precipitated with 0.8 × volume isopropanol, and hooked with a Pasteur pipette into a fresh 1.5-ml eppendorf tube. The hooked DNA was washed with 1-ml 70% ethanol and centrifuged at 8,000 rpm for 5 min at room temperature using a microcentrifuge. The supernatant was decanted, and the tubes air-dried. The isolated DNA was dissolved in 500 μl of 0.1 × Tris–HCl (pH 8.0), and 1.5 μl RNAse A (20 mg/ml stock) was added to each tube and incubated at 37°C for 30 min. An equal volume (500 μl) of Chloroform: Isoamyl alcohol (CI, 24:1) was further added and thoroughly mixed by gentle inversion several times. Centrifugation was carried out at 8,000 rpm for 10 min at room temperature and the supernatant carefully transferred to fresh tubes. DNA was precipitated by adding 2× vol of ice-cold absolute ethanol, and the tubes mixed well (by several inversions) and placed at −20°C for 1 h or overnight. The DNA was hooked with a Pasteur pipette into 1.5-ml eppendorf tube and washed with 1-ml 70% ethanol; and centrifuged at 7,000 rpm for 5 min at room temperature. The supernatant was removed, and the DNA air-dried and resuspended in 100 ul of 0.1 × Tris–HCl (pH 8.0). DNA concentration and quality were determined using a Nanodrop spectrophotometer and further confirmed by agarose gel electrophoresis.

**Table 1:** List of plant materials used for the amplification and isolation of MITE fragments and study of the distribution of MITE insertions and evolutionary relationships in the *Triticum–Aegilops* complex.

Species/genome	Genotype or accession	Abbreviation
*T. aestivum*, 2*n* = 6x = 42, AABBDD	Chinese Spring	CS
*T. aestivum*, 2*n* = 6x = 42, AABBDD	Norin 61	N61
*T. aestivum*, 2*n* = 6x = 42, AABBDD	Synthetic hexaploid wheat ABD. No.4	SHW ABD4
T. aestivum^*^, 2*n* = 6x = 42, AABBDD	Synthetic 72	Syn72
T. aestivum^*^, 2*n* = 6x = 42, AABBDD	Multiple synthetic derivative Original #1	MSD-original #1
T. aestivum^*^, 2*n* = 6x = 42, AABBDD	MSD—2 (Waxless subpopulation)	MSD-2 waxless
T. aestivum^*^, 2*n* = 6x = 42, AABBDD	MSD—5 (Heat-tolerant subpopulation)	MSD-5 heat-tolerant
T. aestivum^*^, 2*n* = 6x = 42, AABBDD	Cytoplasmic substitution line 3–1	Cyto subst. line3-1
*T. turgidum* ssp. *carthricum*, 2*n* = 4x = 28, AABB	34H188, KU-138	T. tur
*T. durum*, 2*n* = 4x = 28, AABB	Langdon	Langdon
*T. dicoccoides*, 2*n* = 4x =28, AABB	KU-110	-
*T. urartu*, 2*n* = 2x = 14, AA	KU-199-1	-
*T. urartu*, 2*n* = 2x = 14, AA	KU-199-10	-
*Ae. tauschii,* 2*n* = 2x = 14, DD	34H203, KU-20-2	Ae. tau.
*Ae. aucheri*, 2*n* = 2x = 14, SS	KU-1-3	-
*Ae. speltoides*, 2*n* = 2x = 14, SS	KU-12962	-
*Ae. speltoides*, 2*n* = 2x = 14, SS	KU-14602	-

* These plants are artificially produced experimental materials having the same genomes of *T. aestivum*.

### PCR amplification of MITE fragments

A total of 52 primer pairs designed from flanking sequences surrounding intact MITEs (Yaakov et al., 2012) were used for the amplification of MITE fragments in this study (see Supplementary Table S1). PCR for amplification of MITE fragments was performed in a total reaction volume of 25 μl, containing 12.5 μl PCR Master Mix (Promega), 1.0 μl of genomic DNA (~50 ng/μl), 1.25 μl of each site-specific primer (6.1 pmol/μl) and 9.0 μl MilliQ water. The PCR was performed in a thermal cycler (GeneAmp PCR System 9,700, Applied Biosystems) using touchdown annealing temperature conditions as follows: initial denaturation at 94°C for 3 min; then 35 cycles with annealing decreasing by 2°C: 5 cycles of 94°C for 1 min, 60°C for 1 min, 72°C for 90 s; 5 cycles of 94°C for 1 min, 58°C for 1 min, 72°C for 90 s; 5 cycles of 94°C for1 min, 56°C for 1 min, 72°C for 90 s; followed by 20 cycles of 94°C for 1 min, 54°C for 1 min, 72°C for 90 s; and a final extension step at 72°C for 4 min. MITEs amplicons were size-separated using 1.5% agarose (Nippon gene, Japan) gel at 100 V for 25 min and stained with ethidium bromide. Stained gels were visualized under UV light and photographed using a gel documentation system.

### Genotyping of RILs with polymorphic MITE fragments

Preliminary genotyping of PCR-SCAR MITE markers in a wheat recombinant inbred lines (RILs) mapping population and its parental genotypes (CS and SHW ABD4) was performed using five polymorphic primers from four *Stowaway*-like MITE families: *Thalos*, *Athos*, *Minos*, and *Eos*). PCR for this analysis was carried out using a total reaction volume of 11.0 μl containing 5.5 μl PCR Master Mix, 4.0 μl of genomic DNA (~3.125 ng/μl), 0.3 μl of each polymorphic MITE primer (6.1 pmol/μl), and 0.9 μl MilliQ water. The thermal cycling conditions were as described above, and amplicons were separated on 1.5% agarose gel at 100 V for 25 min and documented as described above. The easily scorable bands were analyzed and integrated into our wheat genetic linkage map.

### Determining MITEs excision frequency

MITEs excision was assessed in 129 plants of *T. aestivum* cv. CS by PCR using 16 polymorphic CS-specific insertion sites from 9 MITEs families: Thal-EU835982, Thal-EU835981, Thal-CQ169689; Fort-AY663392, Fort-EU835980; Atho-AM932680, Atho-AB201447, Atho-DQ517494; Oleu-AF325198; Mino-FN564434; Eos-FN564434; Pan-DQ871219; Pan-FN564434; Phoebus-102; Polyphemus-110, and Polyphemus-111 (see Supplementary Table S1 for details of these primer sequences). PCR was carried out in a total reaction volume of 13 μl containing 6.5 μl PCR Master Mix, 2.0 μl of genomic DNA (~12.5 ng/μl), 0.625 μl of each polymorphic insertion site - primer (6.1 pmol/μl) and 2.25 μl MilliQ water. The thermal cycling conditions were as described above, and amplicons were separated on 1.5% agarose gel at 100 V for 25 min and documented as described above.

### Statistical analysis

Analysis of similarity (ANOSIM) was conducted to confirm the statistical differences between the different genome types and ploidy levels. Phylogenetic analysis was performed based on Jaccard similarity and then a group average hierarchical clustering was conducted based on a SIMPROF test with 99,999 simulations (alpha < 0.05) using PRIMER6 (PRIMER-E).^1^ Furthermore, a principal component analysis (PCA) was conducted on the similarity to statistically reveal the degree of dissimilarity between hexaploids, tetraploids, and diploids.

## Results

### Amplification of MITE fragments and their isolation in wheat and related species

Five genotypes, CS, SHW ABD4, Norin 61, 34H188, and 34H203 (Table 1) were initially used to detect the presence/absence of MITE fragments and facilitate their isolation *via* PCR. Of the 52 tested MITE primer pairs selected from 13 *Stowaway*-like families (Yaakov et al., 2012; Supplementary Table S1), 48 primer pairs produced amplified products in at least one of the tested genotypes, while four primer pairs did not yield amplified products in any of the tested genotypes which suggested a lack of insertion sites. As has been previously reported (Yaakov et al., 2012), the majority of amplified MITE sequences used in this study were from the B genome (59%, i.e., 17 of the 29 MITE sequences with known chromosomal location), while 12 of the 29 MITE sequences were from the A (6 MITE sequences, 21%) and D (6 MITE sequences, 21%) genomes. The DNA fragments produced from the 48 amplified PCR-SCAR MITE markers contained DNA sequences from 13 *Stowaway*-like MITE families (Yaakov et al., 2012; Supplementary Table S1), as shown in Table 2.

**Table 2:** Number of PCR–SCAR MITE markers obtained from 13 Stowaway-like MITE families.

S/No.	Stowaway-like MTE family	Approximate size (bp)	No. of PCR-SCAR MITE markers obtained
1	Thalos	164	8
2	Fortuna	327	4
3	Athos	85	6
4	Oleus	152	7
5	Minos	240	4
6	Eos	353	3
7	Pan	127	3
8	Aison	219	1
9	Icarus	112	2
10	Phoebus	322	4
11	Polyphemus	232	3
12	Victor	276	1
13	Xados	116	2
Total			48

**Figure 1:**
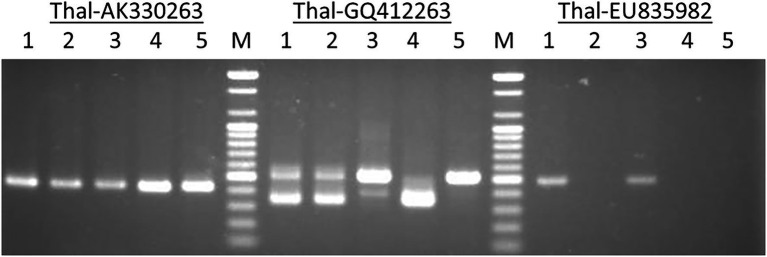
An example of a site-specific PCR for *Thalos* (Thal-GQ169689) that was inserted in the 11th intron of the *plastid glutamine synthetase* 2 (*GS2*) gene in five accessions. The expected “full site,” i.e., the larger band of ~519 bp was found only in the hexaploid Chinese Spring and Norin 61 (Lanes 1 and 3). The Synthetic hexaploid wheat (Lane 2) along with the tetraploid *T. turgidum* (Lane 4) and the diploid *Ae. tauschii* (Lane 5) lacked the full insertion site and only the “empty site,” i.e., the lower band of ~367 bp was found.

**Figure 2:**
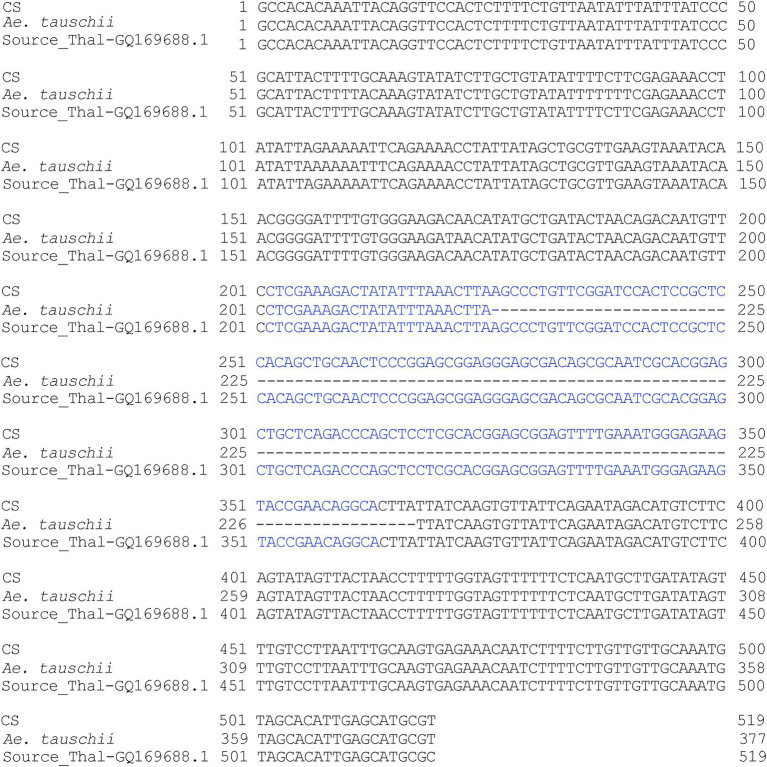
Multiple sequence alignment of sequenced amplified fragments (see Figure 3) corresponding to *Triticum aestivum* cv. Chinese Spring, *Ae. tauschii* and the best match NCBI database (Thal-GQ169688) bread wheat sequence. The *Thalos* element is indicated in blue letters, while the flanking sequences are indicated in black letters. The two *T. aestivum* cultivars Chinese Spring and the NCBI database (bread wheat) have the element, while the *Ae. tauschii* accession lacked it.

**Figure 3:**
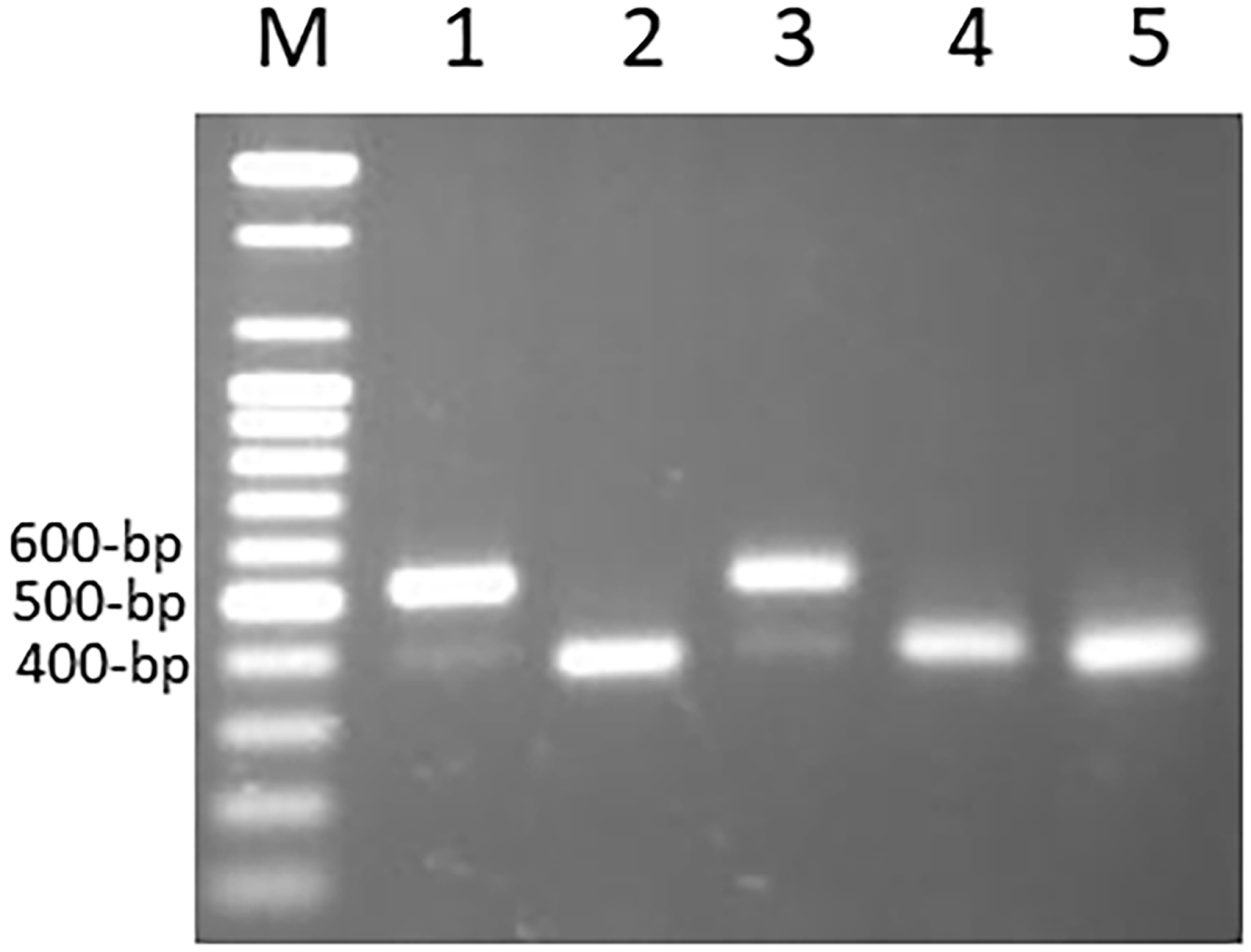
An example of amplified MITE fragments observed in five *Triticum–Aegilops* accessions with three MITE primers (Thal-AK330263, Thal-GQ412263, andThal-EU835982). The five accessions are: (1) Chinese Spring; (2) SHW ABD 4; (3) Norin 61; (4) *T. turgidum*; (5) *Ae. tauschii.*

The primer pairs used in this study were designed by Yaakov et al. (2012) to amplify the MITE insertions and their flanking host sequences to produce the expected full amplicon size, which is termed “full site” (i.e., the size of the MITE insertion plus the flanking sequences), compared to an “empty site,” i.e., without a MITE insertion, in which the amplicon will be relatively shorter in size, consisting of only the flanking sequence. An example of a site-specific PCR for *Thalos* (Thal-GQ169689 in Supplementary Table S1), which was inserted in the 11**th** intron of the *plastid glutamine synthetase* 2 (*GS2*) gene, is shown in Figure 1. In this case (Figure 1), the expected **“**full site**”** is 519 bp-long, while the **“**empty site**”** is 367 bp-long. While the hexaploids CS and N61 had the **“**full site**”** fragment containing the MITE insertion, ABD 4, *T. durum* and *Ae tauschii* lacked the MITE insertion. Very faint bands corresponding to the size of the empty site were observed in the hexaploid species (CS and N61), which could be a footprint probably due to the loss of the fragment in a small percentage of the cells in the tissue. Sequence comparison of the isolated site-specific PCR fragment from CS (“full site”), *Ae tauschii* (“empty site”) and a database sequence (bread wheat, Thal-GQ169688) confirmed that the fragment differences in the gel are due to the presence or absence of this *Thalos* element (Figure 2.). Other examples of MITE amplified fragments observed in this study are shown in Figure 3.

### Distribution of MITEs in 17 accessions of wheat and related species and evolutionary relationships inferred by MITE insertion polymorphisms

**Figure 4:**
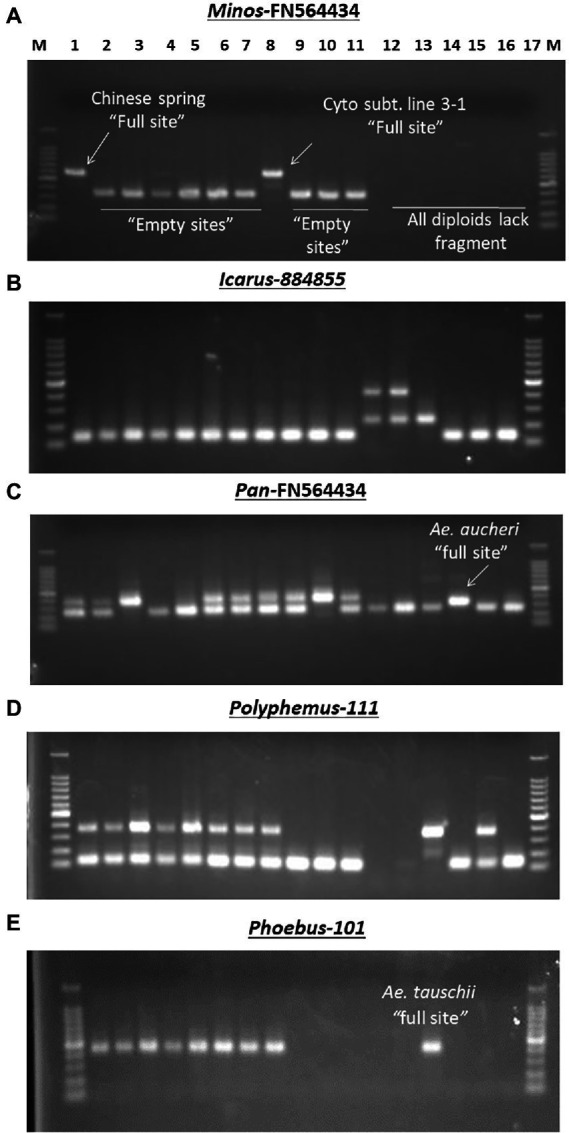
Examples of different MITE insertion polymorphism patterns observed in 17 accessions of wheat and related species in relation to ploidy and/or genome types. **(A)** Amplification patterns observed with *Minos*-FN564434: in this case, only the hexaploid cv. Chinese Spring (Lane 1) and its Cytoplasmic substitution line 3–1 (lane 8) had the *Minos* element, termed as “full site”; the rest of the hexaploid accessions and the tetraploids had only the “empty site,” while all the diploids (lanes 12–17) showed band absence. **(B)** Amplification patterns observed with *Icarus-*884,855: all the hexaploid and tetraploid accessions (lanes 1–11) and the SS diploid types (lanes 15–17) lacked the *Icarus* element with only the “empty site” present; in addition to the Icarus element present in *Ae. tauschii* (DD, lane 14), the two *T. urartu* (AA, lanes 12 and 13) possessed an additional ~450 bp-long unique fragment. **(C)** Amplification patterns observed with *Pan*–FN564434; all the hexaploid and tetraploid accessions [lanes 1–11, except Norin 61 and MSD-Original #1 (lanes 4 and 5, respectively)] had the *Pan* element; while all the diploids (lanes 12–17) except *Ae. aucheri* diploid accession (KU-1-3, lane 15) showed band absence. The relatively lower fragment position of the *Pan* element in KU-1-3 seems to suggest some sequence deletion in this accession. **(D)** Amplification patterns observed with Polyphemus-111; all the hexaploid accessions (lanes 1–8), *Ae. tauschii* (lane 14) and an accession of *Ae. speltoides* (lane 16) had the *Polyphemus* element; the tetraploid accessions (lanes 9–11) and *Ae. aucheri* (lane 15) had only the empty site, while the two *T. urartu* accessions (lanes 12 and 13) accessions showed band absence. **(E)** Amplification patterns observed with Phoebus 101; all the hexaploid accessions (lanes 1–8), along with the diploid *Ae. tauschii* (lane 14) had the *Phoebus* element; the tetraploid accessions (lanes 9–11) and the other diploid accessions lacked the element.

To assess MITE dynamics and determine whether the proliferation of MITEs was of recent origin in allohexaploid wheat, 17 accessions of wheat and related species comprising hexaploids, tetraploids and their diploid progenitors were genotyped with the 43 MITE primer pairs. Of the 43 markers studied, five (12%) were monomorphic, indicating that they may be of fossil origin. Different patterns of MITE insertion polymorphisms in relation to ploidy and/or genome type were also observed (Figure 4). MITE insertion numbers (i.e., abundance) increased with ploidy level (Table 3). The total number of amplified “full sites” ranged from 26 [in SHW ABD4 (AABBDD)] to 37 [in CS (AABBDD) and its cytoplasmic substitution line 3-1(AABBDD)], 22 [in 34H188 (*T. turgidum*, AABB) and Langdon (*T. durum*, AABB)] to 23 [in KU-110 (*T. dicoccoides*, AABB)], and 10 [in KU-1-3 (*Ae. aucheri*, SS)] to 18 [in KU-199-1 and KU-199-10 (*T. urartu*, AA); and 34H203 (KU-20-2, *Ae. tauschii*, DD)] as shown in Table 3. Significant variation in *“full site”* fragments was found among the hexaploids and the BB genome group of the diploids, unlike the AABB and AA/DD genome groups which showed little or no variation in full sites (Table 3). The proportion of polymorphic bands among these accessions ranged from 44 to 76% in *Ae. speltoides* (KU-14602) and *T. aestivum* cv. CS, respectively. An analysis of similarity (ANOSIM) was conducted to further investigate differences at the genomes or polyploid levels. ANOSIM revealed a high degree of dissimilarity between some hexaploids, tetraploids and diploids (*p* < 5% and R > 0.75). A high MITE proliferation was observed in allohexaploid bread wheat (32.6 ± 3.8 insertion sites) relative to the tetraploid (22.3 ± 0.6 sites) and diploid (15.0 ± 3.5 sites) progenitors, which lends support to the notion that rapid activation of transposons occurred recently after the formation of wheat.

**Table 3:** Distribution of amplified MITE fragments in 17 accessions of wheat and related species.

	No. amplified fragments			No. polymorphic fragments		
Genotype	Total sites	Full sites	Empty sites	^a^Total sites	^b^Full sites	^c^Empty sites
**A: Hexaploids**						
Chinese spring	48	37 (77.1%)	11 (22.9%)	41 (85.4%)	31 (75.6%)	10 (24.4%)
Synthetic hexaploid wheat ABD. No.4	39	26 (66.7%)	13 (33.3%)	32 (82. %)	20 (62.5%)	12 (37.5%)
Synthetic 72	41	29 (70.7%)	12 (29.3%)	34 (82.9%)	23 (67.7%)	11 (32.4%)
Norin 61	44	32 (72.7%)	12 (27.3%)	37 (84.1%)	26 (70.3%)	11 (32.4%)
Multiple synthetic derivative Original #1	44	32 (72.7%)	12 (27.3%)	37 (84.1%)	26 (70.3%)	11 (29.7%)
MSD—2 (Waxless subpopulation)	48	34 (70.8%)	14 (29.2%)	40 (83.3%)	28 (70.0%)	13 (30.0%)
MSD—5 (Heat-tolerant subpopulation)	46	34 (73.9%)	12 (26.1%)	39 (84.8%)	28 (71.8%)	11 (28.2%)
Cytoplasmic substitution line 3–1	49	37 (75.5%)	12 (24.5%)	42 (85.7%)	31 (73.8%)	11 (26.2%)
**B: Tetraploids**						
34H188, KU-138	35	22 (62.9%)	13 (37.1%)	28 (80.0%)	16 (57.1%)	12 (42.9%)
Langdon	33	22 (66.7%)	11 (33.3%)	26 (78.8%)	16 (61.5%)	10 (38.5%)
KU-110	35	23 (65.7%)	12 (34.3%)	28 (80.0%)	17 (60.7%)	11 (39.3%)
**C: Diploids**						
KU-199-1	25	18 (72.0%)	7 (28.0%)	18 (72.0%)	12 (66.7%)	6 (33.3%)
KU-199-10	25	18 (72.0%)	7 (28.0%)	18 (72.0%)	12 (66.7%)	6 (33.3%)
34H203, KU-20-2	26	18 (69.2%)	8 (30.8%)	19 (73.1%)	12 (63.2%)	7 (36.8%)
KU-1-3	16	10 (62.5%)	6 (37.5%)	9 (56.3%)	4 (44.4%)	5 (55.6%)
KU-12962	24	14 (58.3%)	10 (41.7%)	16 (66.7%)	8 (50.0%)	8 (50.0%)
KU-14602	23	12 (52.2%)	11 (47.8%)	16 (69.6%)	7 (43.8%)	9 (56.2%)
Total sites scored (43 primers)	64	46	18	56	41	15
**Mean distribution by ploidy**						
Hexploids	44.9 ± 3.6	32.6 ± 3.8	12.3 ± 0.9	37.8 ± 3.5	26.6 ± 3.8	11.3 ± 0.9
Tetraploids	34.3 ± 1.2	22.3 ± 0.6	12.0 ± 1.0	27.3 ± 1.2	16.3 ± 0.6	11.0 ± 1.0
Diploids	23.2 ± 3.7	15.0 ± 3.5	8.2 ± 1.9	16.0 ± 3.6	9.2 ± 3.4	6.8 ± 1.5

a Values in parenthesis indicate the percentage of total polymorphic sites relative to total amplified sites.

b Values in parenthesis indicate the percentage of polymorphic “full” sites relative to the total polymorphic sites.

c Values in parenthesis indicate the percentage of polymorphic “empty” sites relative to the total polymorphic sites.

The observed promising amplification of the MITE fragments in the subset of five accessions (as indicated above) enabled us to subsequently extend a survey of the MITE insertion polymorphisms in a relatively larger set of accessions of wheat and related species shown in Table 1; and different patterns of MITE insertion polymorphisms observed in the 17 accessions of wheat and related species of ploidy and/or genome types shown in Figure 4. Figure 4B, for instance, showed a ~450 bp-long fragment unique to the two *T. urartu* accessions, which might possibly be due to element dimer, which are known to form rapidly during periods of active transposition (McGurk and Barbash, 2018), thereby leading to site duplication in *T. urartu.*

**Figure 5:**
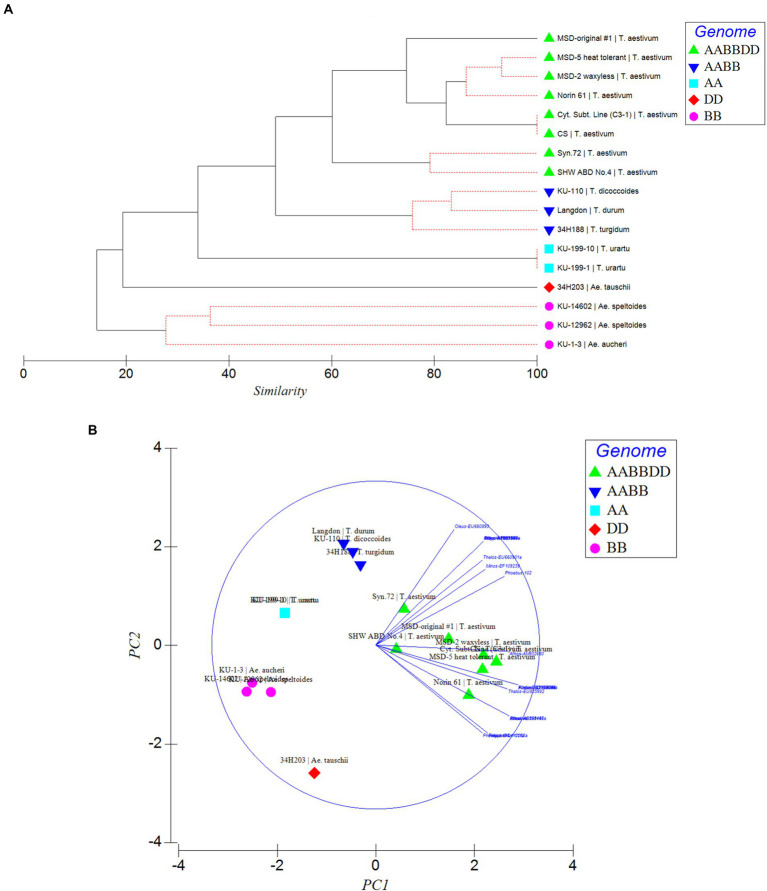
Phylogenetic relationships **(A)** and Principal component analysis (PCA; **B**) inferred by MITE insertion polymorphisms in the *Triticum–Aegilops* complex based on polymorphic “full” sites. Different strata of evolutionary relationships were inferred according to ploidy and genome types.

To further confirm the suitability of the MITE markers to explain the polymorphism between the different accessions tested, phylogenetic trees were produced and PCA analysis was conducted based on the polymorphic “full sites” (Figures 5A,B), which revealed different strata of evolutionary relationships in the *Triticum*–*Aegilops* complex. Generally, the accessions were grouped in five groups in relation to their ploidy and genome constitution: AA, BB or SS, DD, AABB, AABBDD (Figures 5A,B). Within the hexaploids group (Group 1), an original accession of multiple synthetic derivatives was neatly separated from the other accessions including its MSD-2 waxless and MSD-5 heat-tolerant offshoots that are in sub-cluster along with Norin 61, CS and its cytoplasmic substitution line 3–1 (Figures 5A,B). The synthetics were closer to the tetraploids from which they were re-synthesized in Group 2. Group 3 is made up only of diploid accessions comprising of three distantly separated sub-clades in relation to their genome constitution: *Ae tauschii* (DD), *T. urartu* (AA) and *Ae. aucheri* and *Ae. speltoides* (SS). Among the diploid progenitors, our results indicated that, *Ae. tauschii* (DD) is more genetically distant from the other diploid species, followed by *T. urartu* (AA) and then the SS or BB genome type (*Ae. aucheri* and *Ae. speltoides*, which were sub-clustered together; Figures 5A,B). The principal component analysis clearly revealed the relationship among the accessions in relation to their genome groups and ploidy; with the first two principal components explaining 59.7% of the total variation (Figure 5B; Supplementary Table S2). The genomic distribution, as well as the genetic relationships detected by these MITEs insertion polymorphisms, is consistent with the known evolutionary history and phylogenetic relationships in wheat.

### Stability of MITE insertions in the genome of wheat cv. CS

As stable inheritance of transposable elements is a desirable feature for their usefulness as a valuable DNA marker system, we investigated the possible excision of MITEs in the wheat genome using CS, one of the parents of our mapping population. As shown in Table 3, the highest frequency of polymorphic MITE insertion sites was observed in this cultivar. With 16 polymorphic MITE markers ×129 plants amounting to a total of 2,064 sites investigated for possible excision, no single excision was observed in this wheat accession, i.e., the frequency of insertion stability was 100% (data not shown). This observation indicates the value of these MITEs as valuable molecular markers in wheat.

### MITE insertion polymorphism rate in parents of our mapping cross, preliminary genotyping in a RIL population, and linkage mapping

Of a total of 48 amplified MITE markers assayed, 15 primer pairs yielded polymorphic bands between the two parents of our intraspecific mapping cross (CS and ABD4), indicating a 31% polymorphism rate. Of these polymorphic primer pairs, 10 and four showed presence/absence and co-dominant inheritance, respectively, while one primer pair produced both presence/absence/co-dominant fragments between the two parents. Preliminary genotyping using five polymorphic PCR-SCAR MITE primers in our mapping cross-population of 104 RILs yielded six scorable bands with five showing the expected 1:1 segregation ratio for simplex markers, while one of the two markers scored from Athos-DQ5176494 with an approximate size of 277 bp fitted a 3:1 ratio for a duplex **×** nulliplex marker (Table 4). An example of the segregation of MITE markers in a RIL mapping population is shown in Supplementary Figure 1. Interestingly, a high frequency of simplex alleles (83%) was observed with these MITE markers, which indicates the potential usefulness of these stable MITEs for efficient mapping of this complex allohexaploid genome. Four of the six scorable MITE markers obtained for the preliminary genotyping of a RIL mapping population (Table 4) have already been integrated into our current DArT-and SNP-based linkage mapping constructed. The reported chromosomal location of these tested markers was confirmed by our linkage mapping effort, while the location of a hitherto unassigned MITE marker [TE 30–2, Minos-EF567062] which mapped to chromosome 5D was established from our linkage mapping studies.

**Table 4:** MITE marker identity, size, chromosomal location, and segregation ratio tested in a Chinese Spring (P_1_) × SHW ABD No.4 (P_2_) RIL mapping population.

Marker ID	Size (bp)	Location	N^ǂ^	No. present	No. absent	Genetic ratio tested
TE 9–1 (Thal-GQ169689)	598	Chr. 2D	104	52	52	1:1, χ^2^ = 0.000
TE 15–1 (Atho-DQ5176494)	356	Chr. 3B	100	56	44	1:1, χ^2^ = 1.440
TE 15–2 (Atho-DQ5176494)	277	Chr. 3BL	103	77	26	3:1, χ^2^ = 0.115
TE 29–1 (Mino-FN564434)	579	Chr. 3B	103	55	48	1:1, χ^2^ = 0.476
TE 30–2 (Mino-EF567062)	559	Chr. 5D^*^	102	42	60	1:1, χ^2^ = 3.177
TE 34–1 (Eos-FN564434)	822	Chr. 3B	101	55	46	1:1, χ^2^ = 0.802

ǂ Number of plants scored.

* Newly assigned chromosomal location from this study.

## Discussion

We utilized a set of published MITE markers from 13 *Stowaway*-like families, whose main advantage is derived from the presence or absence of a small-sized element that can be easily assayed (Shirasawa et al., 2012; Yaakov et al., 2012) for genomic studies in wheat and related species. The simple inheritance of MITEs, their relatively inexpensive and co-dominant assay method, as well as their relatively high polymorphism rate (even between closely related taxa) make MITE markers highly promising for exploitation in different aspects of genomic studies. The potential of MITE insertion polymorphisms as an efficient DNA marker system has been demonstrated in several plant species including wheat (Yaakov et al., 2012, 2013), groundnut (Shirasawa et al., 2012; Gayathri et al., 2018), rice (Monden et al., 2009), barley (Lyons et al., 2008) and *Brassica* species (Sampath et al., 2014). The MITE markers tested in this study have been reportedly shown to be in association with genes or coding sequences with insertions occurring in introns of known genes, repetitive regions or in intergenic regions (Yaakov et al., 2012; Supplementary Table S1). In this study, similarly to the previous report in wheat by Yaakov et al. (2012), we found these MITE insertional polymorphisms as highly efficient evolutionary markers suited for inferring evolutionary relationships in the *Triticum–Aegilops* complex, genome analysis and linkage mapping in wheat.

MITEs have been known to be found often in close proximity to or within gene-rich euchromatic regions, where they might alter the expression and function of the associated gene(s) (Jiang et al., 2004; Naito et al., 2009). An *in-silico* study by Sabot et al. (2005) showed that ~43% of the MITE insertions occurred in association with wheat genes. Moreover, Yaakov et al. (2012) found 60% of the wheat MITEs used in this study to be associated with genes, with ~51% of the insertions occurring in the introns (Supplementary Table S1). Such insertions of active MITEs in gene-rich regions might drive the evolution of novel genes in wheat, thereby leading to altered phenotypes. For instance, Gorafi et al. (2016) showed a MITE insertion in the promoter region of *Vrn-1A* allele annulled the vernalization requirement of a wheat introgression line. Dai et al. (2021) revealed significant marker-trait associations for five agronomically important traits uncovered by 10 polymorphic markers generated from six MITE specific primer pairs in a diversity panel of 126 *Brassica napus* genotypes. A recent study on the variability of root system architecture in five subspecies of Spanish *T. turgidum* L. revealed differences in unique MITE insertion in the *TtDro1B* gene useful for the reliable differentiation of the subspecies *turgidum* from the *durum* and *polonicum* types (González et al., 2021). Thus, further genetic studies with these MITE markers will provide insights for uncovering useful variation in the genes associated with the activity of these MITE insertions (as shown in Supplementary Table S1) for the molecular breeding of wheat.

The distribution of MITE insertions in relation to different ploidy and genome types (Table 3), revealed the activity of MITEs during evolution. The large difference in observed MITE insertion sites among the hexaploids (26–37 insertion sites), tetraploids (22–23 insertion sites) and their diploid progenitors (10–18 insertions sites), as well as between the diploid genome types (e.g., 10–14 full sites in the SS versus 18 full sites in the AA and DD genome types) which suggest that MITEs might have undergone a recent rapid activation in wheat following the allopolyploidization events. This observation is consistent with the recent report of Keidar-Friedman et al. based on whole genome analysis where the distribution of a total of 239,126 retrieved MITE insertions were found to be 48.2% (in hexaploids), 32,7% (in tetraploid T. turgidum) and 9.6% (in diploids, av. from *T. urartu* and *Ae. tauschii*). Based on the abundance of *Stowaway-like* MITEs in wheat group 7 chromosomes, where more of the 2026 MITEs analyzed were found in 7D (35.79%) relative to the 7A (28.87%) and 7B (35.24%), Lu et al. (2014) suggested that the A and B sub-genomes might have eliminated some repetitive elements during the double hybridization events in allohexaploid wheat. Our present study also revealed more *Stowaway-like* MITE insertions in the D sub-genome relative to the S (or B) sub-genome, but similar numbers of insertion sites were found between the D and A sub-genomes. However, a *de novo* search for MITEs of the entire assembled wheat genome v2 using the MITETracker software enabled the discovery of 6,013 MITE families in the wheat genome, with the MITEs distributed along the chromosomes and associated with gene-rich regions (Crescente et al., 2018). Of the 125,800 different MITEs discovered across the wheat genome based on the MITETracker, the B sub-genome was more MITEs-rich (40.14%) followed by the A sub-genome (32.81%) with the D sub-genome (27.05%) being the least (Crescente et al., 2018). It seems that this situation of the relative abundance of MITEs accords with the general tendency of TEs in allohexaploid wheat, as Wicker et al. (2022) analyzed long terminal repeat (LTR) retrotransposons (full-length) and found the B sub-genome to be relatively more abundant in the TEs studied, followed by the A sub-genome and then D sub-genome. In future studies, it would be worthwhile to clarify the evolutionary consequences of the relative abundance of MITE insertions in the different sub-genomes in allohexaploid wheat and the potential implications of the likely altered function of associated genes in future wheat breeding.

Phylogenetic and PCA analysis based on the detected MITE insertion site polymorphisms revealed high genetic divergence that clearly classified the accessions in the *Triticum–Aegilops* complex consistent with the known evolutionary history of wheat, and sub-grouped the different accessions according to their specific genome types (SS, AA, DD, AABB, AABBDD). This study showed the efficiency of these evolutionary markers in producing high-resolution phylogenetic trees that sub-grouped the accessions according to their specific genome types, consistent with the findings of Yaakov et al. (2012), thereby lending further support to the hypothesis that MITEs were recently active and proliferated in a species-unique fashion. Our observation indicated MITE activity in recently synthesized allohexaploid wheat (including the multiple synthetic derivatives, MSD; Table 1) high polymorphisms in MITE insertion sites especially between the MSD (32–34 sites), a primary synthetic wheat allohexaploid (Syn. 72, 29 sites) and synthetic hexaploid wheat (SHW ABD4, 26 sites). As shown in the phylogenetic tree and PCA (Figures 5A,B), the MSD accessions were clustered in Group 1 with the original MSD line #1 being uniquely separated, while the Syn.72 and ABD4 were sub-clustered in a group close to the tetraploid accessions (Group 2) from which these two accessions were derived. Collectively, our results suggest that MITE transposition activity occurred throughout the course of wheat evolution with rapid activation occurring more recently and might provide further insights in efforts at studying wheat biodiversity and TE-associated gene introgression (Yaakov et al., 2012).

Our study on the stability of the PCR–SCAR MITE markers investigated in this study using *T. aestivum* cv. CS, one of the parents of our mapping population, revealed that they are quite stable in the wheat genome. Similar findings on the stable inheritance of *AhMITE1* (frequency of *de novo* excision = 0.00023, Shirasawa et al., 2012) and the rice *mPing* (0.00023, in 96 EG4 plants under normal conditions, Monden et al., 2009), which indicated their value as excellent molecular markers for linkage analysis, had been reported. No single element excision was found in 129 next-generation cv. CS plants investigated with 16 polymorphic MITE primer pairs, which suggested that element excision will not be a concern in the utility of these markers for linkage analysis in wheat.

Our field observations showed that the cv. CS usually presents stable phenotypes which might explain the lack of observed MITE excision; however, the wheat cv. Norin 33 tends to show a sort of genetic instability (Watanabe, 1962), that may be due to TE activity. As a future strategy, it will be useful to characterize the transposition activity in this known genetically unstable cv. Norin 33 relative to other more stable genotypes such as CS (and possibly SHW ABD4) using the multiplexed transposon display technique or high-throughput sequencing to identify new active transposons or their insertions in wheat.

The PCR-SCAR MITE markers demonstrated effectiveness in detecting insertion length polymorphisms in a CS × SHW ABD4 RIL mapping population developed from an intraspecific cross. The two parental lines were genetically partitioned into main clusters on the dendrogram, thereby suggesting a high degree of genetic divergence between them. The observed MITEs polymorphism rate of 31% detected between the two *T. aestivum* parents highlights the high potential of the MITEs in uncovering polymorphisms for the linkage mapping of bread wheat. Moreover, the stable MITEs showed seemingly simple inheritance and with a very high frequency of simplex markers (83% markers with a 1:1 segregation ratio, Table 3) that are potentially useful for the efficient mapping of the complex allohexaploid wheat. It generally seems that TE-based markers such as those based on insertional polymorphisms detectable by PCR-SCAR primers designed from their conserved flanking sequences or their multiplexed assay derivatives [e.g., MITE transposon display, Sequence-specific amplified polymorphism (SSAP), etc.], have a tendency to generate simplex markers that are even more suitable for the molecular mapping of complex polyploid genomes. A study in sweet potato (Monden et al., 2015) based on *Rtsp*-1 retrotransposon insertion polymorphisms found an abundance of simplex markers (~90%) which enhanced the mapping efficiency of the genetically complex autohexaploid sweet potato. In bent grass, a MITE-display analysis using four selective primer pairs (Amundsen et al., 2011) uncovered a total of 139 polymorphic markers, of which 28 markers fitted the expected 1:1 or 3:1 genetic ratio with the simplex marker types being the most abundant (~81.4%). Based on a RIL population of 104 individuals, four simplex MITE markers developed from our preliminary genotyping effort (TE 29–1, Minos-FN564434–Chr. 3B; TE 34–1, Eos-FN564434–Chr. 3B; TE 15–1, Athos-DQ5176494–Chr. 3B; and TE 30–2, Minos-EF567062–Chr. 5D) were integrated into our current DArT-and SNP-based linkage map being constructed. The reported chromosomal location of Minos-FN564434, Eos-FN564434 and Athos-DQ517494 in chromosome 3B was confirmed by our linkage mapping effort, while the chromosomal location of one of the MITE markers [Minos-EF567062, mapped to chromosome 5D (data not shown)] was, to the best of our knowledge, established for the first time from our linkage mapping studies.

Overall, these MITE markers which are simply inherited and well resolved on short gel runs in 1.5% normal agarose gels, that can be substituted with an automated system to increase the efficiency and reduce time, are very promising as cost-effective markers for exploitation in the genome analysis and evolutionary studies in wheat. Several sequences of these MITE fragments have been used to design transposon display primers to extend the frontiers of this research for the rapid genotyping of the wheat genome. Our preliminary transposon display-based MITE genotyping results using two selective primer pairs (data not shown) generated a large number of bands in the accessions tested. Thus, the MITE markers used in this study are promising and will potentially serve as a robust resource for several applications in wheat genetics and molecular breeding including biodiversity and evolutionary studies, linkage analysis, association mapping and MITE-associated modification of gene expression.

## Data availability statement

The original contributions presented in the study are included in the article/Supplementary material, further inquiries can be directed to the corresponding authors.

## Author contributions

BU, YG, YM, and HT: conceptualization. BU: experimentation. BY, KK, and BU: annotation of sequences and primer design. BU and YM: methodology. BU and YG: data curation. BU, BY, and YG: formal analysis. BY and KK: software. BU, YG, BY, YM, KK, and HT: interpretation of data. BU: writing—original draft. BU, YG, YM, BY, KK, and HT: writing—review and editing. HT: genetic resources, funding acquisition, and project administration. All authors contributed to the article and approved the submitted version.

## Conflict of interest

The authors declare that the research was conducted in the absence of any commercial or financial relationships that could be construed as a potential conflict of interest.

## Publisher’s note

All claims expressed in this article are solely those of the authors and do not necessarily represent those of their affiliated organizations, or those of the publisher, the editors and the reviewers. Any product that may be evaluated in this article, or claim that may be made by its manufacturer, is not guaranteed or endorsed by the publisher.

## Footnotes

^1^ https://onlinelibrary.wiley.com/doi/10.1111/j.1442-9993.1993.tb00438.x

## Supplementary material

SUPPLEMENTARY FIGURE 1 An example of the segregation of a MITE marker (Minos-FN564434) in 104 Chinese Spring (P1) x SHW ABD. No.4 (P2) RIL mapping population. The genotypes of the two parental lines (CS and SHW) are shown.
