# Supplementary figures and images for: Exploiting the miniature inverted-repeat transposable elements insertion polymorphisms as an efficient DNA marker system for genome analysis and evolutionary studies in wheat and related species
Source: Front Plant Sci. 2022 Sep 2;13:995586. doi: 10.3389/fpls.2022.995586 (PMC9479669; doi:10.3389/fpls.2022.995586)

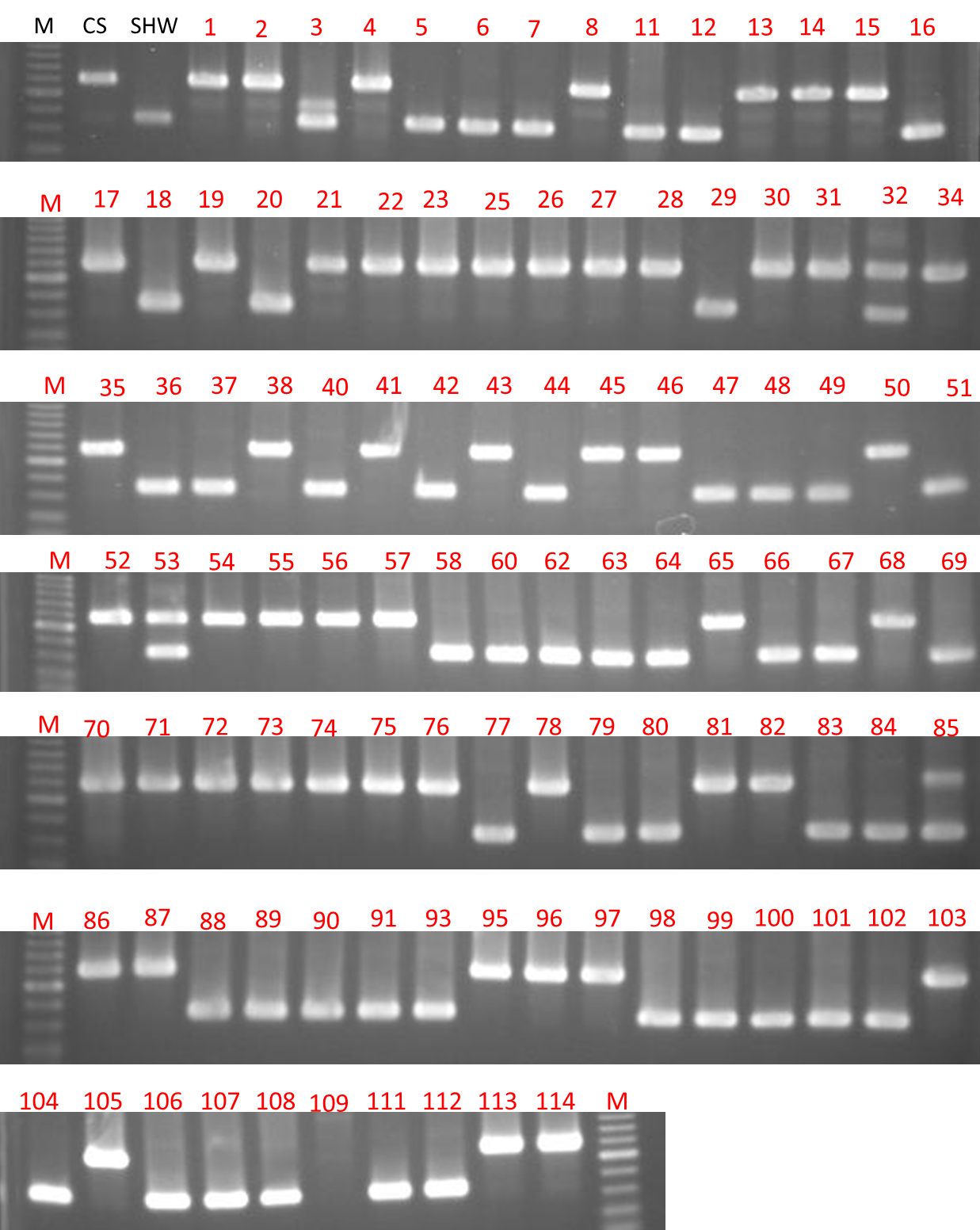

Supplement: SUPPLEMENTARY FIGURE 1 — An example of the segregation of a MITE marker (Minos-FN564434) in 104 Chinese Spring (P1) x SHW ABD. No.4 (P2) RIL mapping population. The genotypes of the two parental lines (CS and SHW) are shown. [file Image_1.tif]
