# Supplementary material for: Exploiting the miniature inverted-repeat transposable elements insertion polymorphisms as an efficient DNA marker system for genome analysis and evolutionary studies in wheat and related species
Source: Front Plant Sci. 2022 Sep 2;13:995586. doi: 10.3389/fpls.2022.995586 (PMC9479669; doi:10.3389/fpls.2022.995586)
Supplement: Supplementary file 2 [file Table_1.pdf]

Table S1: Details of the 52 wheat MITE insertional polymorphism markers used in this study (cf. Yaakov et al. 2012; Beery Yaakov - Unpublished data)§

Transposon name	Marker name*	Primers	Tm(°C)¥	Element size (bp)	Expected "full site" (bp)	Expected "empty site" (bp)	*Source of the sequence and genome /chromosome location (if known)*	MITE location/Annotation (if known)
Thalos	Thal-AK330263	TCAAATCGACTCCTTTTTGG<br>CAACCAGGGCAACTGTGTAG	50.6<br>56	170	421	251	*Ae. tauschii* - from cDNA clone	[Expressed sequence]
	Thal-GQ412263	GAATTGTTGATGATCGAGCTG<br>GTGTCATTGGGGCTGGTAAC	51.8<br>56.1	165	500	335	*Ae. tauschii* (chro.1D)	[9th Intron] ACV41079.1: phosphomannomutase D1
	Thal-EU835982	GCCTCACTTCTTTCGTCGTC<br>GGGACACGTTGTGGTGTATG	55.8<br>56	165	478	313	*T. aestivum (* Chinese Spring, from genome D)	[Intergenic] Between cell division protein AAA ATPase family (Pseudo) and ACJ22517.1: unknown
	Thal-EU835981	TGCAGACAACACCAAGCTCT<br>AACTGGAAGCCTCAAAGCAG	56.9<br>55.5	164	486	322	*T. aestivum (Chinese Spring* , from genome B)	[Intergenic] Between beta-1-3-galactosyl-o-glycosyl-glycoprotein (Pseudo) and ACJ22516.1: hypothetical protein
	Thal-EU660901	TTGCCATGAATGTGGCAGT<br>CATCCACATAGGATGTCTCTCG	55.3<br>54.5	163	400	237	*T. durum* (chro. 2BS)	[Intergenic] ~53kb from ACD46684.1: plastid acetyl-CoA carboxylase
	Thal-AM932686	GTGACCCCCGACAATCATAG<br>CAAGCATATGACTACACACACACA	55.1<br>55.2	164	428	264	*T. aestivum (* Renan, chro.3B)	[Repeat region] Caspar, Egug, Jorge, Democles
	Thal-AY951945**	GGTGCCCATTAAACAGTTGC<br>TTGAACCACAGACCCAAAAA	54.9<br>52.5	165	484	319	*T. monococcum*	[Intergenic] Downstream to AAY32563.1: CRT/DRE binding factor 9
	Thal-AY188331	TGAACAACATGGAGGTGAGAA<br>GTTGACCCCCGACAATCATA	54<br>54.6	164	448	284	*T. monococcum (* DV92, chro.5AL)	[Intergenic] ~45kb from AAO72630.1: MADS box transcription factor AP1
	Thal-GQ169689	GCCACACAAATTACAGGTTCC<br>ACGCATGCTCAATGTGCTAC	54.4<br>56	152	519	367	*T. aestivum* (Chinese Spring, chro. 2D)	[11th Intron] ACT22500.1: plastid glutamine synthetase 2
Fortuna	Fort-AF326781	GAGAGGACGACGGTGGTTTA<br>GCAGCGATTTAGGATTGGAC	56.4<br>53.9	343	694	351	*T. monococcum*	[Repeat region]
	Fort-AY663391	GGCGGTGGTTTAAGATGAGA<br>GCAGCGATTTAGGATTGGAC	54.8<br>53.9	343	686	343	*T. turgidum (* Langdon)	[Intergenic] ~23kb from AAW78913.1: putative resistance protein
	Fort-AY663392	AACAAGTCATGGCATGTCAAAG<br>CATGCCACATTGTTGATCG	54<br>52.1	315	667	352	*T. aestivum (* Renan)	[Intergenic] Upstream to AAW78917.1: nodulin-like protein
	Fort-EU835980	GGAAAAGTTCATGCATTTGG<br>GTGATGTAAAACTCCAACTCCACA	50.3<br>55.2	305	598	293	*T. aestivum (Chinese Spring* , from genome A)	[Intergenic] ~11kb from ACJ22498.1: chalcone synthase
Athos	Atho-DQ537336	GGAGCACAGGATAGAGCTGAA<br>AGGGGCGAAGCTGTATTTTT	56.3<br>54.8	85	333	248	*T. aestivum (Chinese Spring* , chro. 1B)	[Intergenic] downstream of ABG68033.1: receptor kinase 2
	Atho-DQ517494	TGTTTTCGCATCGTCTCTTG<br>TGTTGTTGGGCCAAAATCTG	53.3<br>54	85	356	271	*T. aestivum (* Yongchuan white wheat, chro. 3B)	[3rd Intron] ABF48530.1: VIVIPAROUS1 protein
	Atho-FJ393539	TGCGAATCATGGCAAATAGA<br>CCTCCTGCACCATACTGAAC	52.3<br>55.3	85	400	315	*T. turgidum* ssp. *dicoccon* (chro. 7BL)	[2nd Intron] ACQ59150.1: phytoene synthase 1
	Atho-AM932680	CTGAGACGATTTAGCGTGACC<br>GTTGCATCAAGTGCATCCAG	55.4<br>54.6	85	400	315	*T. aestivum (Chinese Spring* , chro. 3B)	[Repeat region] between Laura (LTR) and Caspar (DNA)
	Atho-AB201447	AGGTGGTCTGGGAGATGTTG<br>TTCCTTCACTGATGGTGTGG	56.6<br>54.7	85	390	305	*Ae. tauschii* (chro. 7D)	[3rd Intron] *Triticum aestivum* wSSII-D gene for starch synthase II-D
	Atho-AF029897	ATCAGCTCCGGAAGTGGATA<br>GTCATGTGGGTACGGGTTTC	55.3<br>55.8	85	432	347	*T. aestivum (* TAM-107)	[2nd Intron] *Triticum aestivum* acetyl-coenzyme A carboxylase (Acc-1,1) gene, nuclear gene encoding plastid protein
	Atho-AY368673**	TGGGCAATTCTAATGTTAGCC<br>GGCCCCGTACTGCAGATATT	53<br>57	84	392	308	*T. turgidum* (chro. 1B)	[Intergenic] next to AAQ93631.1: receptor protein kinase
Oleus	Oleu-AF325198	CAAAGCATACAGTCGACCTTTTC<br>TGGCAGACAGTCTCATGGAA	54.2<br>56	153	457	304	*Ae. tauschii* (chro. 1D)	[1st Intron] AAK20743.1: LRK14
	Oleu-EU660893	CATGCATACTAGTCCAAAAGCA<br>TTTGGATTCCGGTGCTAGTT	53.1<br>54.2	154	721	567	*T. aestivum* (Chinese Spring, chro. 3AL)	[Intergenic] ~30kb from ACD46670.1: cytosolic acetyl-CoA carboxylase
	Oleu-FN564436	CGATCCCATTCCTTGATCTG<br>AGCATTCGCTAACAAGCACA	52.9<br>55.2	155	499	344	*T. aestivum (* Chinese spring, chro. 3B)	[7th Introm] CBH32667.1: Transcription factor S-II domain containing protein, expressed
	Oleu-AB298186	AGGCACATCAACCCATTGTT<br>CATGCACCATGTTCGATAGAG	55.1<br>53.5	159	697	538	*Ae. tauschii (* chro. 5DS)	[Intergenic] ~6kb from BAF97103.1: P450 and ~5kb from BAF97104.1: P450
	Oleu-FN564430	TGCTCTAATGCTGCGCTTAC<br>GTTGCAACAGGAACACATGC	55.5<br>55.3	153	468	315	*T. aestivum (* Chinese spring, chro.3B)	[5th Intron] CBH32562.1: VAMP-like protein, putative, expressed
	Oleu-AY951944	AATGAGCATCGCAGCAAAC<br>AGCGATGCCTGCTTTTCTAA	54.2<br>54.7	144	398	254	*T. aestivum (Chinese Spring* , chro. 5A)	[Intergenic] ~3kb from AAY32556.1: CRT/DRE binding factor 15
	Oleu-EU835198**	GCATCGAACTCACTGCAAAA<br>GAAATTCGCCAAAATGTGGT	53.9<br>52	155	462	307	*T. turgidum ssp. dicoccoides Langdon* (chro. 6B)	[7th Intron] ACF33182.1: wheat kinase-START domain protein
	Oleu-FN564428	TTTGTGTGTAGCTTTGCAGGA<br>GAAAATACGGTCGGGTGAAG	54.9<br>53.5	146	398	252	*T. aestivum* (Chinese spring, chro. 3B)	[Repeat region] Upstream to TAA_ctg0091b.t00120.1
Minos	Mino-FN564434	CCTGGAGAAGGACCTGAACT<br>GAGCGAGACCCTTTGACATC	56.1<br>55.5	239	579	340	*T. aestivum* (Chinese spring, chro. 3B)	[Intergenic] ~10kb from CBH32638.1: conserved hypothetical protein, Hv-pg4 homolog,expressed
	Mino-EF567062	TGTCCAAAAGACAAAGCCGTA<br>CCTGGCGCGTAGAGAGATAA	54.7<br>56.3	241	559	318	Ae. tauschii [Chr. 5D]	[Intergenic] ~4kb from ABU54404.1: disease resistance protein (Lr1)
	Mino-EF109239	ATGCAGTGGTTGCTTGTCAT<br>GCTCCCCATCGTTAGGAAAT	55.3<br>54.7	238	561	323	*T. aestivum (* Chinese spring, chro. 3BL)	[1st Intron] ABO31821.1: malate dehydrogenase (Partial)
	Mino-GQ169685	CATGGCATGCACCACTATTA<br>GCATTTTCTTGTCAGCAGCA	52.9<br>54.2	241	560	319	*T. durum* (chro. 2A)	[3rd intron] ACT22495.1: plastid glutamine synthetase 2

**Eos**	Eos-AY494981	TGCTGAGCCCCTAACAGAAC<br>CAGCAGTAATGAGCCGTCAA	57.1<br>55.2	354	839	485	*T. turgidum* (chro. 1A)	[Intergenic] ~50kb from putative receptor kinase (Pseudo)
	Eos-FN564434	GCTGATCAAAGTCTTGGGATTC<br>AACCTTCCACGTGTTTCCAC	53.8<br>55.6	354	822	468	*T. aestivum* (Chinese spring, chro. 3B)	[Repeat region] Near TAA_ctg0954b.00460.1
	Eos-DQ871219	GTTTGGTGACATGCGTGAAC<br>TCTCTGTCAAACCGCCTACC	54.9<br>56.7	351	629	278	*T. turgidum* ssp. dicoccoides Langdon (Chro. 6B)	[Repeat region] ~35kb from ABI15897.1: putative rhamnogalacturonate lyase
**Pan**	Pan-FN564427	AATTTGGCACCAGAGCAAAG<br>CCGTCCTGGTTTACTCTTCC	54.1<br>55	127	444	317	*T. aestivum* (Chinese spring, chro. 3B)	[Repeat region]
	Pan-DQ871219	TTCTCTCCGGTCTCCACGTA<br>GACCCGAGTAGCATGTAGCA	57.1<br>56.5	127	452	325	*T. aestivum* (Chinese Spring, chro. 6B)	[Intergenic] Upstream to ABI15899.1: marker Xucw84
	Pan-FN564434	GAATGCAACACCGCACTACA<br>GTCATCACCCATCAACCACA	55.9<br>55	127	382	255	*T. aestivum* (Chinese spring, chro. 3B)	[1st Intron] CBH32653.1: Oxidoreductase NAD-binding domain
**Aison**	Aison-623445	TGCACGAGAATTTGGTTCTG<br>GACAGCACAGTGCACAAGGT	53.4<br>58.2	219	500	281	*T. aestivum* (Chinese Spring, Chro. 3B)	[Repeat protein] ABB78080.1: powdery mildew resistance protein, PM3CS*
**Icarus**	Icarus - 884855	TCCGTCCGAAAATACCTGTC<br>CGACCAAGAGACCGGTTAGA	54.4<br>56.1	112	334	222	ND	
	Icarus - 4131124	AATGTGGTTGCATGTTGCAT<br>ACGAGTAGGGCGTCTCACAG	53.8<br>58.7	112	348	236	*T. aestivum* (Chinese spring, chro. 3B)	-
**Phoebus**	Phoebus-101	TCTGTTGTCTCAATTGAATAAAGTCTC<br>CGCTAGCCCACATCGAAA	53.6<br>55.2	319	421	102	*T. aestivum* (Chinese spring, chro. 3B)	
	Phoebus-102	AGGCGTTGTTGTAATGTTCTTTT<br>CTCCCTCCTATCCTTTCTAGTTTACA	53.6<br>55.2	319	419	100	*T. aestivum* (Chinese Spring, chro. 3B)	
	Phoebus-103	TCGTTGAGAGTGTGCAGTGA<br>CGAGCCATGCAATTCAGTAA	56.2<br>53.3	319	431	112	*Ae tauschii* (Chro. 1DS)	-
	Phoebus-104	GCCGAAACAGCACTTCCTAT<br>GGAATGGTCCGAAGGTAAAGA	55.3<br>54.3	330	382	52	ND	
**Polyphemus**	Polyphemus-109	CGAAAGTGGTAGGGACGAAA<br>CACGTTAATTCAATGTGAGGACA	54.6<br>53.4	227	412	185	*T. aestivum* (Chinese spring, chro. 3B)	
	Polyphemus-110	CCTAATTGACGTATGTAATGGGTTT<br>GGGTAAAACGTTAAGGGCAAG	53.2<br>54.1	239	357	118	*T. aestivum* (Chinese Spring, Chro. 3B)	-
	Polyphemus-111	TCGAGCTTACTGAAACAATCCA<br>GGTCACTCACCACATGGACA	54.1<br>57	241	389	148	*T. aestivum* (Chinese spring, chro. 3B)	
	Polyphemus-112**	CAACGGTTTGCTAGAGCCTAA<br>ATGGATGCAATCGGTTCAGT	55<br>54.6	220	350	130	ND	
**Victor**	Victor - 120	CACGGTTCATGCATGGAGTA<br>GGACGGCATTGTTTAGAAGC	55<br>54.3	276	498	222	*T. aestivum* (Chinese Spring, Chro. 3B)	-
**Xados**	Xados - 118	CGTTTAGAGAAAGGCGAAGG<br>CAAAGGAGACACAACCGCTAT	53.4<br>55	116	305	189	*T. aestivum* (Chinese Spring, Chro. 3B)	
	Xados - 119	TGTGCAAACACGGACTGATT<br>GGTTTCTTTGCTGCTTCGTC	55.1<br>54.7	116	360	244	ND	-

Footnotes: * from annotation of Aison sequence from TREP database (http://wheat.pw.usda.gov/ITMI/Repeats/nrTREP_list.html); **No amplification; ¥ Tm valuesof ordered primers are slightly lower from Yaakov et al. 2012; § expected size of element, full and empty sites fragment is as per Yaakov et al. 2012 and/or as confirmed in this study; - = Not Known; ND =not determined
