# Supplementary material for: Exploiting the miniature inverted-repeat transposable elements insertion polymorphisms as an efficient DNA marker system for genome analysis and evolutionary studies in wheat and related species
Source: Front Plant Sci. 2022 Sep 2;13:995586. doi: 10.3389/fpls.2022.995586 (PMC9479669; doi:10.3389/fpls.2022.995586)
Supplement: Supplementary file 3 [file Table_2.pdf]

Table S2. Eigenvalues and proportion of variation explained by the principal components analyzed in the 17 *Triticum-Aegilops* species with differing genomic constitution using polymorphic MITE markers

PCs	Eigenvalues	Proportion of variation (%)	Cumulative variation (%)
1	3.41	42.2	42.2
2	1.41	17.5	59.7
3	0.847	10.5	70.2
4	0.698	8.6	78.8
5	0.475	5.9	84.7
